# Biomarkers of Inflammation and Immune Function and Risk of Colorectal Cancer

**DOI:** 10.1007/s11888-015-0282-5

**Published:** 2015-07-23

**Authors:** Alicia Garcia-Anguita, Artemisia Kakourou, Konstantinos K. Tsilidis

**Affiliations:** Department of Hygiene and Epidemiology, University of Ioannina School of Medicine, Stavros Niarchos Av., University Campus, Ioannina, Greece; Department of Epidemiology and Biostatistics, School of Public Health, Imperial College London, London, UK

**Keywords:** Inflammation, Colorectal cancer, Biomarkers, C-reactive protein, Interleukin 6, Tumor necrosis factor α, Genetic polymorphisms, Cohort studies, Risk of bias

## Abstract

A substantial number of prospective epidemiological studies have been conducted to investigate the association between biomarkers of inflammation and immune function and risk of colorectal cancer. Although pre-diagnostic concentrations of these biomarkers, especially C-reactive protein, have been associated with a higher risk of colorectal cancer in some studies, this association does not seem to have a robust support without hints of bias. Future prospective studies should evaluate multiple inflammatory biomarkers with longitudinal measures over the follow-up taking advantage of new multiplex cytokine quantification arrays and use more sophisticated joint or biomarker pattern statistical approaches to capture the complex and dynamic interplay between biomarkers and risk of colorectal cancer. Large collaborative consortia and Mendelian randomization studies should be encouraged to diminish the threat of biases and improve the reliability of this literature.

## Introduction

Colorectal cancer (CRC) is the third most commonly diagnosed cancer worldwide that accounted for 1.36 million new cases and 694,000 deaths in 2012 [1]. CRC develops through a gradual accumulation of genetic, both inherited and somatic, and epigenetic changes, leading to the transformation of normal colonic mucosa into invasive cancer. Approximately 70 to 90 % of CRCs arise from adenomatous polyps (adenomas) [2]. The risk of CRC increases with age especially after the age of 50 years, and the risk is also increased by certain high-penetrance inherited genetic mutations (familial adenomatous polyposis and hereditary non-polyposis colorectal cancer), several low penetrance mutations [3], a personal or family history of colorectal neoplasia, or a personal history of inflammatory bowel diseases (IBD) [4]. Several modifiable factors are also associated with increased risk of CRC, including obesity, physical inactivity, smoking, heavy alcohol consumption, type II diabetes, and a diet high in red or processed meat, while the use of aspirin or other non-steroidal anti-inflammatory drugs (NSAIDs) lowers risk [5].

Systemic inflammation is a pathway through which several of the aforementioned risk factors may lead to colorectal neoplasia. For example, obesity is associated with a state of chronic inflammation induced by excessive production of storage lipids and high circulating concentrations of glucose, both of which create a proinflammatory oxidative environment that has been associated with colorectal carcinogenesis [6, 7]. Adipocytes constitutively express the proinflammatory cytokine tumor necrosis factor α (TNF-α), and TNF-α expression in adipocytes is markedly increased in obese rodents [8].

However, the link between inflammation and colorectal neoplasia is not only substantiated at a systemic level, and it has been further hypothesized that sporadic colorectal neoplasia arises from colonic areas with chronic subclinical inflammation. Several lines of evidence implicate chronic inflammation as a key predisposing factor to colorectal cancer in IBD [9]. First, the risk for developing colorectal cancer increases with longer duration of colitis. Second, the more colonic surface is involved with colitis, the greater the colon cancer risk. Third, evidence is mounting to suggest that anti-inflammatory medications, especially 5-aminosalicylates, can reduce the development of colorectal dysplasia and cancer in IBD. The evidence directly implicating colonic inflammation as a key predisposing factor to sporadic colorectal neoplasia is weaker compared to the evidence for colitis-associated colorectal neoplasia. Some, but not all, clinical studies have shown that colorectal adenoma and cancer patients had higher fecal calprotectin levels, a direct measure of gastrointestinal mucosal inflammation, than normal subjects [10–12]. Colonic microbiota are being increasingly recognized as important contributors to the health of the gastrointestinal tract and may not only lead to CRC development via modulation of the gut barrier function and initiation of inflammation at the colonic epithelium but could also promote systemic inflammation via leakage of bacterial endotoxins into the circulation [13, 14].

The aim of this review is to examine and critically appraise the associations in the literature between biomarkers of inflammation and immune function and risk of CRC and through which infer about the strength of the evidence linking systemic and/or chronic low-grade colorectal inflammation to colorectal carcinogenesis.

## Circulating Concentrations of Inflammatory Biomarkers and Risk of CRC

### C-Reactive Protein

C-reactive protein (CRP) is a sensitive but non-specific systemic marker of inflammation that is produced mainly in the liver in response to cytokines released by phagocytes during infection, trauma, surgery, burns, tissue infarction, advanced cancer, and chronic inflammatory conditions [15]. The circulating concentrations of CRP in healthy individuals are low, usually below 1 mg/L, but they sharply increase in acute infection reaching concentrations well above 10 mg/L. The half-life of CRP is 19 h [16].

A total of 18 prospective epidemiological studies have been conducted to date to investigate the association of pre-diagnostic high-sensitivity CRP concentrations with risk of CRC (Table 1) and have been summarized in three meta-analyses [25, 37, 38•]. Fourteen out of 18 studies included in the most recent meta-analysis showed a higher risk of CRC with higher concentrations of CRP, but only six of them yielded statistically significant results [38•]. The summary random effects relative risk of CRC per one unit change in the natural logarithm (ln) of CRP was 1.12 (95 % CI, 1.05–1.21), but substantial heterogeneity was evident among the studies (*P* heterogeneity, 0.006; *I*^2^, 52 %) [38•]. The summary association was statistically significant for colon cancer (RR, 1.13; 95 % CI, 1.05–1.21) but not for rectal cancer (RR, 1.03; 95 % CI, 0.90–1.17), although there was not a statistically significant difference between the estimates by anatomical location. Moreover, the summary association for CRP and CRC appeared stronger in men (RR, 1.18; 95 % CI, 1.06–1.30) than in women (RR, 1.05; 95 % CI, 0.96–1.16), but again the confidence intervals of the associations by sex overlapped. The results of this meta-analysis were consistent to the results of prior meta-analyses, including the one from our group [37].

**Table 1:** Characteristics of studies investigating the association between circulating concentrations of inflammatory biomarkers (per one unit change in the ln-transformed concentrations of CRP, IL-6, and TNF-α) and the risk of colorectal cancer

Author, year	Country	Population	Study design	No. of cases	No. of controls/population	Inflammatory biomarkers	RR (95 % CI)
Erlinger, 2004 [17]	USA	CLUE II	NCC	172	342	CRP	Colorectum 1.27 (1.03–1.57) Colon 1.37 (1.08–1.74) Rectum 0.99 (0.61–1.59)
Zhang, 2005 [18]	USA	WHS	Cohort	169	27,913	CRP	Colorectum 0.89 (0.76–1.04) Colon 0.80 (0.39–1.67) Rectum 0.52 (0.28–0.99)
Ito, 2005 [19]	Japan	JACC	NCC	141	327	CRP	Colorectum 1.11 (0.94–1.31) Colon 1.11 (0.90–1.37) Rectum 1.13 (0.86–1.49)
Il’yasova, 2005 [20]	USA	HABCS	Cohort	41	2438	CRP IL-6 TNF-α	Colorectum 1.44 (1.03–2.02) Colorectum 1.44 (0.90–2.31) Colorectum 0.90 (0.42–1.94)
Trichopoulos, 2006 [21]	Greece	EPIC-Greece	NCC	48	996	CRP	Colorectum 1.19 (0.81–1.74) Colon 1.32 (0.84–2.08) Rectum 1.41 (0.94–2.11)
Siemes, 2006 [22]	Netherlands	Rotterdam Study	Cohort	189	6273	CRP	Colorectum 1.04 (0.88–1.22) Colon 1.15 (0.71–1.88) Rectum 0.70 (0.50–0.99)
Otani, 2006 [23]	Japan	JPHC	NCC	375	750	CRP	Colorectum 1.11 (0.95–1.30) Colon 1.06 (0.87–1.29) Rectum 1.19 (0.90–1.58)
Gunter, 2006 [24]	Finland	ATBC	NCC	130	260	CRP	Colorectum 1.33 (1.05–1.69) Colon 1.32 (0.97–1.79) Rectum 1.49 (1.02–2.17)
Heikkila, 2009 [25]	UK	BWHHS	Cohort	32	3274	CRP IL-6	Colorectum 0.97 (0.70–1.34) Colorectum 0.92 (0.53–1.60)
Heikkila, 2009 [25]	UK	Caerphilly	Cohort	41	1144	CRP IL-6	Colorectum 0.89 (0.66–1.22) Colorectum 0.71 (0.41–1.23)
Allin, 2009 [27]	Denmark	CCHS	Cohort	191	10,082	CRP	Colorectum 1.52 (0.86–2.67)
Aleksandrova, 2010 [28]	Europe	EPIC	NCC	1096	1096	CRP	Colorectum 1.06 (0.99–1.13) Colon 1.09 (1.01–1.18) Rectum 0.99 (0.88–1.10)
Chan, 2011 [29]	USA	NHS	NCC	280	555	CRP IL-6	Colorectum 0.85 (0.67–1.09) Colorectum 1.09 (0.77–1.55)
Lee, 2011 [30]	Korea	Health exam records	Cohort	158	80,781	CRP	Colorectum 1.59 (1.13–2.22)
Prizment, 2011 [31]	USA	ARIC	Cohort	166	9836	CRP	Colorectum 1.36 (1.06–1.75) Colon 1.38 (1.05–1.82) Rectum 1.12 (0.64–2.00)
Van Hemelrijck, 2011 [32]	Sweden	AMORIS	Cohort	480	102,749	CRP	Colon 1.00 (0.92–1.07)
Ho, 2012 [33]	USA	WHI-OS	NCC	457	841	IL-6 TNF-α	Colorectum 0.91 (0.60–1.38) Colorectum 0.77 (0.51–1.17)^a^
Toriola, 2013 [34]	USA	WHI-OS	NCC	988	988	CRP	Colorectum 1.09 (0.97–1.24) Colon 1.13 (0.99–1.30) Rectum 0.86 (0.62–1.20)
Song, 2013 [35]	USA	HPFS	NCC	274	532	CRP IL-6	Colorectum 1.25 (0.94–1.67) Colon 0.97 (0.68–1.39) Rectum 0.88 (0.49–1.56) Colorectum 1.45 (1.05–2.00)
Wu, 2013 [36]	China	SMHS	NCC	288	576	CRP	Colorectum 1.65 (1.10–2.48) Colon 1.90 (1.12–3.23) Rectum 1.29 (0.66–2.54)

*CRP* C-reactive protein; *IL*-*6* interleukin-6; *TNF*-*α* tumor necrosis factor alpha; *NCC* nested case-control study; *RR* relative risk; *WHS* Women’s Health Study; *JACC* Japan Collaborative Cohort Study; *JPHC* The Japan Public Health Center-based Prospective Study; *ATBC* Alpha-Tocopherol Beta-Carotene Cancer Prevention Study; *CCHS* Copenhagen City Heart Study; *EPIC* European Prospective Investigation into Cancer and Nutrition; *AMORIS* Apolipoprotein Mortality RISk study; *ARIC* Atherosclerosis Risk in Communities study; *NHS* Nurses’ Health Study; *BWHHS* British Women’s Heart and Health Study; *HPFS* Health Professionals Follow-up Study; *HABCS* Health, Aging and Body Composition cohort; *WHI*-*OS* Women’s Health Initiative Observational Study; *SMHS* Shanghai Men’s Health Study

^a^Association reported for top vs. bottom quartile

The first prospective study was published in 2004 from the CLUE II cohort of Washington County, MD, with 172 CRC cases and 342 matched controls and found a twofold statistically significant increase in the risk of colorectal cancer comparing persons with the highest versus lowest fourth of C-reactive protein [17]. A subsequent study by Zhang and colleagues was performed within the Women’s Health Study, which recruited female health professionals, and found an almost statistically significant inverse association between CRP and colorectal cancer [18]. The two largest studies in the field were published from the European Prospective Investigation into Cancer and Nutrition (EPIC) in 2010 and the Women’s Health Initiative observational cohort in 2013 with approximately 1000 CRC cases each, but observed weak and borderline statistically significant associations [28, 34]. A new study was published in 2014, not included in the published meta-analyses, using the NHANES III data and found an imprecise but statistically significant increased risk of colorectal cancer mortality comparing CRP above 1 mg/dL versus undetectable concentrations, but the study did not use a high-sensitivity CRP assay and observed only 59 CRC deaths [39].

The evidence linking CRP concentrations with colorectal adenoma development, a precursor in the natural history of colorectal neoplasia, is weaker. Pre-diagnostic CRP concentrations were not associated with colorectal adenoma in our study in the CLUE II cohort [40]. A subsequent case-control study nested in the Prostate, Lung, Colorectal, and Ovarian Cancer Screening Trial observed a statistically significant 15 % reduction in risk per unit increase in ln CRP [41], which could reflect an attempt by the host to suppress tumor formation. Other studies observed mixed results from null association in a case-control study in Hawaii to a higher risk of large adenomas for participants in the highest category of CRP concentrations in a case-control study in Japan [26, 42].

Given that high-sensitivity CRP is a circulating marker for inflammation, the lack of a consistent association with colorectal neoplasia raises questions on its use as a marker of the inflammatory process associated with colorectal cancer. When we investigated whether increased CRP is a marker of colonic inflammation, we found no such association in a colonoscopy-based study [43•], which implies that CRP may be marking systemic factors (e.g., obesity) that influence colorectal cancer risk or that the observational associations between CRP and colorectal cancer may be afflicted by biases.

In 2012, our group critically appraised the literature of 98 biomarkers and cancer risk evaluating 847 individual studies to shed light on the existence of potential biases and identify associations with robust evidence for their presence [44••, 45]. We concluded that the association between CRP and CRC was not among the robust associations without suggestion of bias, as evidence of large between-study heterogeneity, small-study effects (i.e., smaller studies tended to give larger estimates of effect size compared with larger studies), and excess statistical significance (i.e., the observed number of studies with nominally statistically significant results was greater than the expected number, calculated based on the statistical power to detect a plausible effect) was identified [44••]. It is thus probable that the statistically significant studies in this field are subject to selective reporting or other biases. Selective reporting bias may emerge when there are many analyses that can be performed (using, for example, different adjustments for confounders or models with different statistical terms for CRP and confounders), but only the analysis with the most statistically significant result is presented [46, 47]. Studies demonstrating an association between inflammatory markers and colorectal neoplasia may also have reverse causation bias due to clinically undetected neoplasia at the time of blood collection. Inadequate control for potential confounders may also influence the findings of observational studies, as obesity, physical activity, and use of aspirin or non-steroidal anti-inflammatory drugs are each strongly related to both inflammatory activity and CRC risk.

Further evidence suggesting a lack of a causal link between CRP and colorectal neoplasia comes from candidate gene association studies that have observed mixed results for the association of genetic variants in the *CRP* gene and colorectal cancer risk [22, 48–52] and genome-wide association studies that have not identified *CRP* variants to be associated with CRC risk [3]. Mendelian randomization analyses have used single-nucleotide polymorphisms in *CRP* and/or genetic scores thereof as instruments of the circulating CRP concentrations in order to correct for potential biases of the observational association and have generally reported null results [50, 53, 54], except for a study in EPIC where genetically twofold higher CRP concentrations were associated with higher risk of CRC, but this result lost nominal statistical significance using alternative definitions of instrumental variables [49]. However, all these Mendelian randomization studies have used suboptimal sample sizes with number of cancer cases below 900 [55]. Future studies should take advantage of recent developments in this field, i.e., the use of efficient designs and methods to adjust for violation of assumptions [56–58], to elucidate whether potential causal associations exist between inflammatory biomarkers and cancer risk.

### Interleukin-6

Interleukin-6 (IL-6) is a multifunctional cytokine produced by a variety of hematopoietic and non-hematopoietic cells [59] and exerts a proinflammatory role by acting either on a transmembrane type 1 cytokine receptor or by binding to a soluble IL-6 receptor (sIL6R). IL-6 upregulates several acute-phase proteins such as CRP, fibrinogen, α1-antitrypsin, and serum amyloid A [60]. There is ample mechanistic evidence suggesting an involvement of IL-6 in CRC development. In vivo experiments on wild-type mice have shown that IL-6 is significantly augmented at the colonic tumor environment, and the growth of colon tumors was suppressed when the mice were treated with antibodies against IL6R [61]. However, results from epidemiological studies regarding the association of circulating IL-6 concentrations and risk of subsequent colorectal cancer development have been sparse, as only six prospective studies exist (Table 1) [20, 25, 29, 33, 35].

II’yasova and colleagues found that serum IL-6 concentrations were not statistically significantly associated with the risk of colorectal cancer in the Health Aging and Body Composition Cohort [20]. Similarly, no association was observed in two prospective studies in the UK published in 2009 [25], but the latter three studies included only 30 to 40 colorectal cancer cases. Chan et al. used 279 cases from the Nurses’ Health Study but also did not observe a statistically significant association [29], and another large publication with 413 cases from the Women’s Health Initiative observational cohort yielded a similar not significant finding [33]. Only a recent paper from the Health Professionals Follow-up Study observed a borderline significant increased risk of colorectal cancer with higher IL-6 concentrations, but this association did not remain significant when the first 2 years of follow-up were excluded [35]. When the results of the latter six studies were synthesized in a meta-analysis, a non-significant summary relative risk was observed (RR, 1.10; 95 % CI, 0.88–1.36) per one unit change in ln IL-6 without substantial between-study heterogeneity (*P* heterogeneity, 0.18; *I*^2^, 35 %) [38•].

The epidemiological studies have not in general reported results by tumor location, except for the Health Professionals Follow-up Study that observed a statistically significant increased risk of colon cancer with higher IL-6 concentrations, but the estimate was attenuated and not statistically significant anymore after excluding cases that occurred within 2 years of the baseline blood draw [35]. No association was observed for rectal cancer risk. Very recently, we finalized an analysis of pre-diagnostic plasma IL-6 concentrations and risk of colorectal cancer in the CLUE II cohort using 173 incident colorectal cancer cases and 345 matched controls. We observed that participants in the highest tertile of IL-6 concentration had a statistically significant and greater than twofold higher risk of colon cancer compared to participants in the bottom tertile (Tsilidis KK, personal communication). This association remained statistically significant after excluding cases that occurred within 2 or 5 years from the start of follow-up.

The association between *IL6* genetic polymorphisms and risk of CRC has been evaluated in several candidate gene studies with largely null results [48, 62–64]. The -174G>C polymorphism (rs1800795) has been widely evaluated, and a recent meta-analysis of 11 studies incorporating 6481 cases and 7935 controls did not find a statistically significant association [65].

### TNF-Alpha

TNF-α is another pleiotropic cytokine that is elevated in response to infection and tissue damage and has been shown to be involved in the pathogenesis of IBD [66]. However, scarce epidemiological evidence exists about a potential association between pre-diagnostic TNF-α concentrations and risk of colorectal cancer (Table 1). No statistically significant associations have been observed in two North American prospective studies [33, 67]. Consistent with this evidence, Vaughn and colleagues reported a null association between TNF-α and colon adenoma in a colonoscopy-based case-control study [68]. A surrogate marker for TNF-α, soluble tumor necrosis factor receptor 2 (sTNFR-2), that is more stable in stored frozen samples and less influenced by diurnal variation was significantly associated with increased risk of CRC in women in the Nurses’ Health Study [29], but no association was observed in two subsequent studies either in women or in men [35, 69]. Concentrations of sTNFR-2 were not associated with colorectal adenoma recurrence in patients with a prior adenoma [70]. Candidate gene association studies have not observed statistically significant findings between SNPs in *TNFa*, especially rs1800629, and risk of colorectal cancer [48, 62–64].

### Other Inflammatory Biomarkers

The literature evidence regarding the association of circulating concentrations of inflammatory biomarkers, other than CRP, IL-6, and TNF-α, and the risk of colorectal cancer is very sparse. Serum amyloid A (SAA) is a non-specific inflammatory marker produced in the liver in response to infection, trauma, and other inflammatory states and has been hypothesized to be a more sensitive marker of inflammation over CRP for certain diseases [15, 71]. However, pre-diagnostic concentrations of SAA were not associated with CRC in the Women’s Health Initiative Observational Study [34]. Macrophage inhibitory cytokine 1 (MIC-1) is another novel inflammatory biomarker, which was positively associated with colorectal cancer risk in the Nurses’ Health Study and the Health Professionals Follow-up Study [72]. Fibrinogen concentrations were positively associated with risk of colon cancer in another prospective study in the USA, but white blood cell count, von Willebrand factor, factor VIII, and albumin did not yield statistically significant associations [31]. However, white blood cell count was associated with an increased risk of colon cancer in a Swedish and a Korean prospective study [32, 73]. A recent nested case-control study in the EPIC cohort studied the association of neopterin, a biomarker of cellular immune activity, with the risk of colorectal cancer and found evidence of a U-shaped association, but neopterin has been shown to exert pleiotropic effects on cellular aging, oxidative stress, and inflammation [74]. Another pleiotropic biomarker, advanced glycation end products (AGE), that accumulates during aging and induces oxidative stress and inflammation has been investigated in the prospective Alpha-Tocopherol, Beta-Carotene Cancer Prevention (ATBC) study. High pre-diagnostic concentrations of AGE were not associated with colorectal cancer risk, but high concentrations of the soluble AGE receptor yielded a lower risk [75].

The adipose tissue is an active endocrine organ that secretes several cytokines and hormones, collectively termed adipokines (e.g., leptin, adiponectin, and resistin), which have been demonstrated to have pro- or anti-inflammatory functions. The association between adipokines and colorectal cancer risk has been investigated in several epidemiological studies, the results of which have been mixed [76]. The description and critical appraisal of this evidence are out of scope for the current review, as these adipokines have many pleiotropic effects (e.g., energy homeostasis, insulin signaling, and vascular reactivity) and cannot be considered primarily inflammatory biomarkers.

Calprotectin is a calcium- and zinc-binding protein released by activated innate immunity cells. It is found in cells, tissues, and fluids throughout the body and has been studied in a variety of inflammatory conditions [77]. Fecal calprotectin has been hypothesized to be a direct measure of gastrointestinal mucosal inflammation [78, 79]. Some clinical studies have shown that colorectal adenoma and cancer patients had higher fecal calprotectin levels than normal subjects, but a recent meta-analysis reported a non-statistically significant association between fecal calprotectin and colorectal neoplasia [12].

## Conclusions and Future Directions

The overall literature evidence for an association between inflammatory biomarkers and risk of colorectal cancer is inconclusive. Although concentrations of inflammatory biomarkers, especially CRP, have been associated with colorectal cancer risk in some studies, this association does not seem to have a robust support without hints of bias (e.g., selective reporting, reverse causation, residual confounding). This does not necessarily mean that these biomarkers are not truly associated with colorectal cancer, but there is still substantial uncertainty about them. If any potential causal association is identified in the future between an inflammatory biomarker and risk of colorectal cancer, then this might lead to the potential development or repurposing of drugs targeting receptors of inflammatory markers.

There are several ways to improve this evidence in the future. First, the measurement and estimation of a single biomarker and colorectal cancer association in most of the existing studies do not consider the complex synergisms among the many inflammatory biomarkers. Recent developments in serum cytokine quantification technology include multiplex arrays that can measure multiple biomarkers in each sample and better evaluate the complex and dynamic nature of inflammatory responses with substantial cost and sample savings [80]. When many biomarkers are available per participant, then a more sophisticated joint or biomarker pattern statistical approach or an exposure-wide association study could be used to assist in capturing the complete picture of the biomarker and cancer association. Some of these methods have been used in the existing literature, but their use is still sparse and their results are inconsistent and imprecise [31, 32, 34, 69, 81]. Second, the single measurement of an inflammatory biomarker in time does not represent an average concentration over time and may lead to regression dilution bias. Future studies should better ascertain how long-term exposure to concentrations of inflammatory biomarkers may be related to colorectal cancer risk, as reports have shown that the long-term stability of inflammatory biomarkers is good but not perfect [82, 83].

Third, large collaborative consortia should be encouraged in this field. The use of standardized definitions and protocols for exposures, outcomes, confounders, and statistical analyses may diminish the threat of biases and improve the reliability of this literature. Many of the biases may be further lessened if studies were more completely and transparently reported according to published guidelines, such as the Strengthening the Reporting of Observational Studies in Epidemiology statement and its extension for Molecular Epidemiology [84–86]. Statistical significance testing should not be used as a criterion, either from authors or editors, for publication of biomarker studies. Future Mendelian randomization studies could also assist in elucidating potential causal associations.
